# Sacrococcygeal Teratoma: Mistreated With Repeated Aspirations

**DOI:** 10.21699/ajcr.v7i3.422

**Published:** 2016-06-15

**Authors:** Ramagopal G, Guru R, Suresh P, Moorthy G, Devi Lu

**Affiliations:** 1 Department of Pediatrics, Chettinad Hospital and Research Institute, Kelambakkam, Chennai; 2 Department of Pediatric Surgery Chettinad Hospital and Research Institute, Kelambakkam, Chennai.

**Dear Sir,**

Here we report a case of mature sacrococcygeal teratoma (SCT) in a small girl which was not diagnosed early and was treated as collection of fluid in gluteal region with recurrent needle aspirations since the age of three months.

One year and nine month old baby girl brought to the hospital with swelling in the left gluteal region noticed by the parents since three months of age. They consulted a local practitioner who suggested aspiration by needle, which was done multiple times. The size of the swelling used to decrease immediately after aspiration but returned to its usual size within 1-2 months. On examination, the child was alert, active with normal growth and development. Left gluteal region was more prominent with skin over the region showing multiple puncture marks (fig. 1), palpation revealed an ill-defined, immobile, and non-tender mass with variable consistency. Per rectal examination showed mass extending up to the presacral region. Bimanual palpation revealed extension of the mass up to the level of the pelvic brim. Ultrasound revealed a well-defined anechoic cystic lesion near coccyx. MRI of the left gluteal region showed two peripherally enhancing lesions near the anterior-inferior part of the coccyx confirming the diagnosis of SCT (fig. 1). Serum alpha fetoprotein level was normal. At operation, a large dumb-bell shaped mass with both solid and cystic (mainly) components, arising from the coccyx, was completely excised along with coccygectomy. Postoperative recovery was uneventful. Biopsy report showed it to be mature teratoma (fig. 1) with margins free of tumor. The parents were counseled for regular follow-up.

**Figure:**
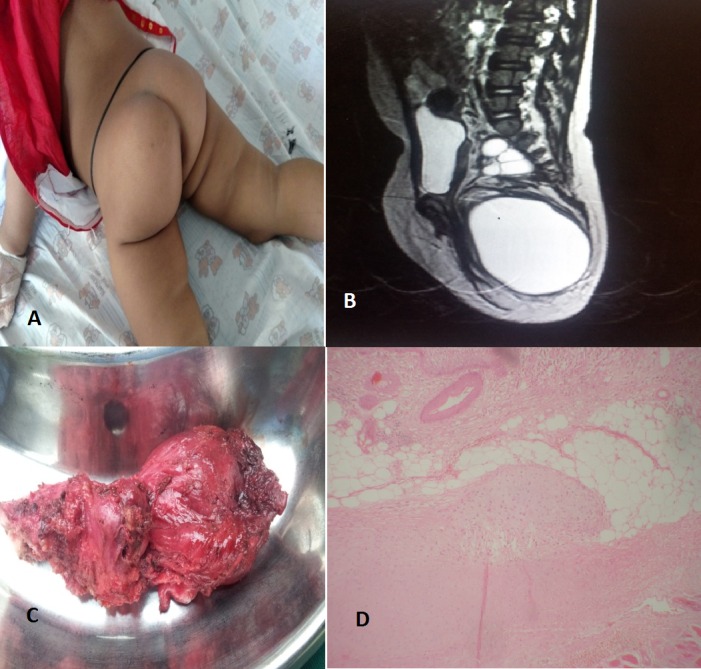
Figure 1: Showing left gluteal swelling (A), MRI (B) showing a cystic mass; excised specimen (C); histopathology showing mature teratoma (D).

SCT is one of the tumors in the neonatal period which carries an excellent prognosis provided timely diagnosis, prompt surgical treatment and complete excision along with coccygectomy are done.[1, 2] The chances of malignancy in SCT relates to factors like the tumor size of >10 cm, Altman Type III and IV tumors, presence of more solid component, and late presentation.[2] Our patient presented late but fortunately the tumor was mainly cystic. Recurrence has been reported in SCT as a benign or malignant tumor especially during the first 3 years of life. Factors like tiny foci of malignant endodermal sinus cells, incomplete resection of tumor, tumor spillage, and omission of coccygectomy increases the possibility of recurrence.[2,3] In our case despite late presentation, the mass was removed completely along with the coccygectomy, and histopathology confirmed it as mature teratoma. Thus a favorable outcome is noted. Awareness programs are needed for general practitioners about common pediatric surgical diseases and their management.

## Footnotes

**Source of Support:** Nil

**Conflict of Interest:** None declared

